# Microglial contributions to aberrant neurogenesis and pathophysiology of epilepsy

**DOI:** 10.20517/2347-8659.2020.02

**Published:** 2020-07-12

**Authors:** Tanya R. Victor, Stella E. Tsirka

**Affiliations:** Department of Pharmacological Sciences, Stony Brook University, Stony Brook, NY 11794, USA.

**Keywords:** Microglia, epilepsy, neurogenesis, neuroinflammation, seizures

## Abstract

Microglia are dynamic cells that constitute the brain’s innate immune system. Recently, research has demonstrated microglial roles beyond immunity, which include homeostatic roles in the central nervous system. The function of microglia is an active area of study, with insights into changes in neurogenesis and synaptic pruning being discovered in both health and disease. In epilepsy, activated microglia contribute to several changes that occur during epileptogenesis. In this review, we focus on the effects of microglia on neurogenesis and synaptic pruning, and discuss the current state of anti-seizure drugs and how they affect microglia during these processes. Our understanding of the role of microglia post-seizure is still limited and may be pivotal in recognizing new therapeutic targets for seizure intervention.

## INTRODUCTION

Epilepsy is a neurological disorder characterized by recurrent seizures. Microglia, the innate immune cells of the central nervous system (CNS), are increasingly recognized as mediators of seizures and contributors to the epileptogenic process. The progression to epilepsy is characterized by the presence of neuroinflammation, as well as structural and molecular alterations in the brain, that subsequently lead to increased neuronal hyperexcitability and a lasting disposition towards spontaneous recurrent seizures (SRS)^[1]^. Microglia regulate neuroinflammation and axonal sprouting and have been reported to modulate neurogenesis. Following seizures, microglia are activated, functioning as resident macrophages of the brain and respond quickly to injury while trying to maintain the physiological processes under its control^[2]^. Changes in neuronal homeostasis are also observed, highlighting the diverse ways in which microglia could be contributing to the development of epilepsy.

This review will discuss the roles of microglia in neuroinflammation and neurogenesis, and how these contributions are altered post-seizure. We will examine microglia in the context of epileptogenesis, the process by which “the previously normal brain is functionally altered and biased towards the generation of abnormal electrical activity that subserves chronic seizures”^[3]^. Additionally, we will explore studies of pharmacological reagents and their effects on microglia as a therapeutic target to mitigate the epileptogenic process that drives epilepsy.

## EPILEPSY

Epilepsy is a chronic brain disorder characterized by abnormal brain activity that causes seizures. The propensity to generate recurrent seizure events has neuropathological, cognitive, and social consequences^[4]^. Epileptic seizures are aberrant, excessive, or synchronous neuronal discharges and manifest in a variety of ways. According to the International League Against Epilepsy (ILAE), seizures are classified into three types based on their onset: generalized onset seizures do not have a determined area of origin and can affect both sides of the brain; focal onset seizures originate from one area of the brain; and unknown onset seizure when the onset is missed or obscured. Generalized onset seizures can present with a variety of manifestations that include non-motor and motor presentations: they range from absence seizures (that present with lapses in awareness, accompanied with staring into space, probably accompanied by rapid blinking and/or orofacial automatisms) to generalized tonic-clonic seizures with tonic and/or clonic spasms, and are always accompanied by loss of consciousness. Focal onset seizures may or may not be accompanied by a loss of awareness and their origin can be attributed to a specific area of the brain that causes motor or sensory changes, including taste or smell. Focal seizures may also result in a loss of awareness, manifested by a person who appears to be dazed, confused, and unable to respond to questions for several minutes. Focal seizures may become generalized if the original behavior, which was localized to one brain hemisphere, expands to behaviors that involve both sides of the brain^[5]^. The cause of epilepsy in many patients is not known, though acquired causes include stroke, traumatic brain injury (TBI), autoimmune disorders, infection, and tumors.

It is estimated that almost 10% of people will experience a seizure in their lifetime^[6]^. Epilepsy affects approximately 1.2% of the population in the United States alone^[7]^. Higher incidence rates have been reported in younger (early childhood and infancy) and older age groups (older than 55 years of age), while a lower prevalence is seen in the period between early adulthood and midlife^[8]^. The imbalance between excitatory and inhibitory neurotransmission (E/I imbalance), with a propensity towards increased excitation, is believed to be the underlying cause of seizures in epilepsy. Research demonstrates hyperexcitability during ictogenesis, when excitatory glutamatergic activity is increased while inhibitory gamma aminobutyric acid (GABA) ergic activity is dampened^[9–11]^. Currently, the treatment of epilepsy varies from patient to patient. Anti-seizure medications are typically the first choice of therapy for subsequent seizure prevention. When medication fails, surgery has been successful in significantly decreasing or making patients seizure free, though only a small number of patients with focal onset seizures would qualify for surgical options^[12]^. When surgery is not an option, patients are treated with antiepileptic drugs (AEDs). There have been > 30 medicines that have been approved by the United States Food and Drug Administration (FDA) or the European Medicines Agency (EMA). Even though many seizure medication options exist, nearly 33% of patients fail to respond to them^[13]^. Some patients with pharmacologically refractory epilepsy try to control seizures by exploring dietary changes, such as employing the ketogenic diet, a high fat/low carbohydrate diet which can be successful in reducing seizures in about 50% of adult patients^[14]^. Though originally believed to result in an increase in levels of GABA production^[15]^, there may be multiple mechanisms that contribute to its success in seizure cessation^[16]^. Neurostimulatory devices, such as deep brain or vagus nerve stimulation therapies, have also been used with varying success, as they help to normalize the excitatory state of the brain^[17]^.

### Epileptogenesis

Epileptogenesis is the process by which structural and molecular changes occur in the brain and predispose towards epileptic seizures^[18]^. The epileptogenic process can be initiated by multiple underlying causes such as tumors, infections, stroke, and brain injuries. Epileptogenesis occurs prior to an unprovoked seizure and continues beyond the event. It is a dynamic process that can occur very quickly, after brain injury or stroke, or over an extended period of time (up to months in animal models, and years in humans)^[18,19]^. This window presents a temporal opportunity for treatment approaches, but also provides challenges for studying the process. Understanding the pathophysiological changes that occur during epileptogenesis is a pivotal part of developing new therapies.

**Figure 1.**
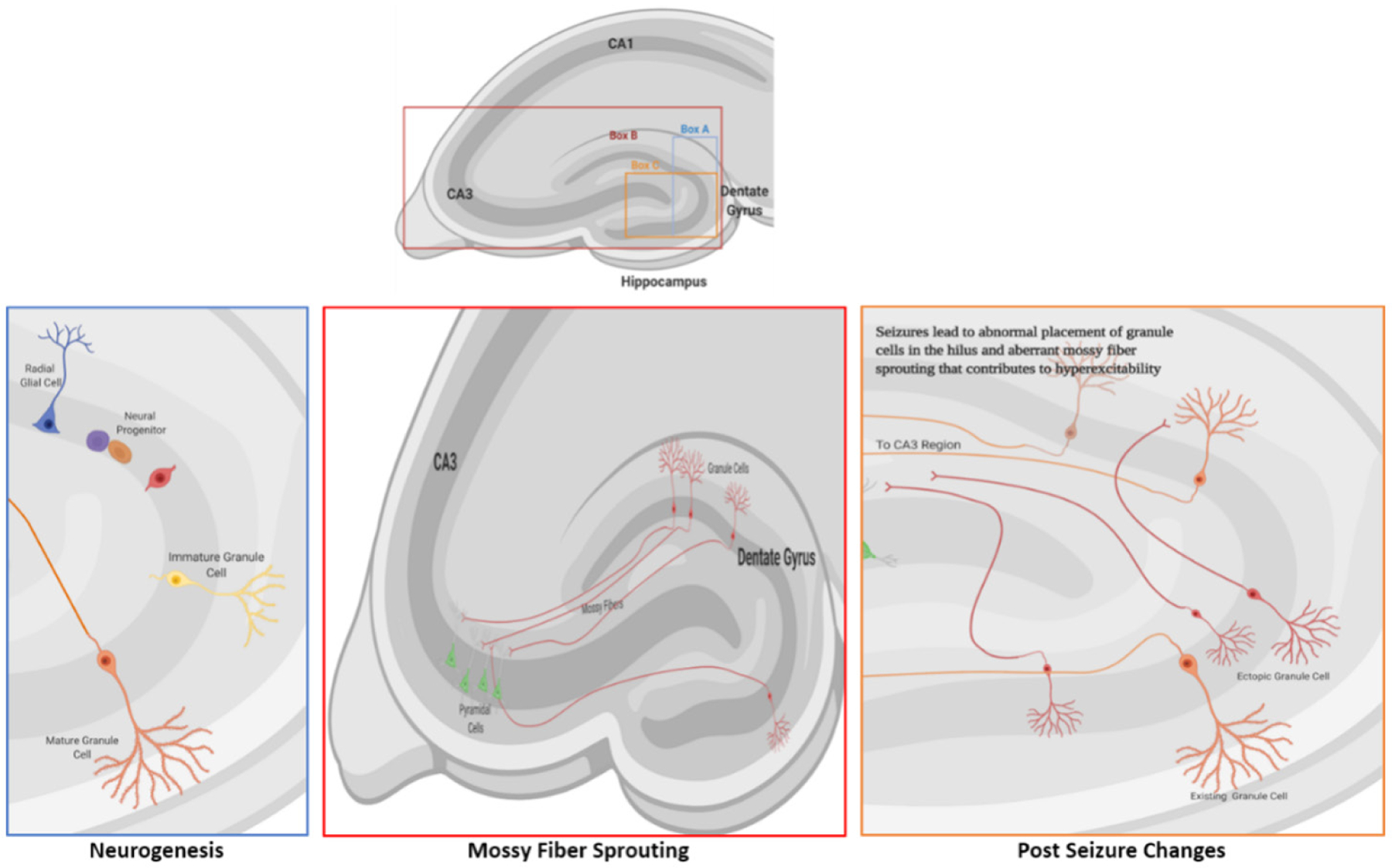
Granule cell neurogenesis and mossy fiber sprouting. A: Neurogenesis occurs in the dentate gyrus of the hippocampus. The cells proliferate in the subgranular zone and then migrate a short distance to the granule cell layer where they differentiate into mature granule cells; B: the axons of granule cells (mossy fibers) normally project to the cells in the CA3 region of the dentate gyrus; C: during seizures, several factors contribute to aberrant migration of granule cells that leads to their ectopic placement in the hilus. Ectopic granule cells (red cells) form functioning neural connections to the pyramidal neurons in the CA3 region and contribute to hyperexcitability and epileptogenesis through aberrant ‘sprouting’ along the mossy fiber pathway. Image created with BioRender.com

Changes during epileptogenesis occur in both neuronal and glial cells, all of which contribute to the dysfunction of neuronal circuits. The mechanisms underlying epileptogenesis suggest that the pathophysiological and compensatory changes are connected. Animal models of epileptogenesis have displayed histologically-detectable changes, such as sprouting along the mossy fiber pathway, neurogenesis, and gliosis [Figure 1] alterations, all of which can contribute to the potential for hyperexcitability^[20]^. The condition most frequently associated with mossy fiber sprouting is temporal lobe epilepsy (TLE), the most common type of epilepsy in adults^[21]^, but can occur in epilepsy patients without TLE^[22]^. Sprouting occurs when granule cell axons in the inner molecular layer (mossy fibers) project into the hilus of the dentate gyrus and CA3 region of the hippocampal formation, creating their own dendritic field. Mossy fibers synapse onto hilar mossy cells, CA3 pyramidal cells, and interneurons^[23]^ to create de novo recurrent excitatory circuits. Aberrant sprouting in a model of TLE was reported to contribute to excitatory feedback loops of normal and ectopic granule cells^[24]^. Another study described aberrant mossy fibers that drive inhibitory basket cells to reduce neuronal excitability^[25]^. Mossy fiber sprouting is increased through the activation of several granule cell factors, such as neuromodulin and brain-derived neurotrophic factor (BDNF)^[26]^, and involves the secretion and deposition of molecules of the extracellular matrix that facilitate aberrant growth^[27–29]^. The number of granule cells also affects mossy fiber sprouting. Hippocampal neurogenesis, which leads to the formation of new granule cells, is increased shortly after an epileptic seizure, but the increase is transient. The development of new granule cells, and their ectopic integration into neuronal networks contribute to aberrant mossy fiber sprouting that is evident post-seizure.

Reactive gliosis has also been identified as a contributor to epileptogenesis in genetic and chemically-induced animal models of epilepsy^[30]^. Activated astrocytes and microglia exhibit changes that promote network hyperexcitability^[31,32]^. Microglia can be activated by cytokines and monocytes circulating in blood^[33]^, neurotransmitters released by activated or damaged neurons, or by molecules migrating across the blood brain barrier (BBB)^[31]^. Disruption of the BBB during status epilepticus (SE) leads to the transport of plasma proteins and immune cells into the brain. The combined effects on astrocytic functions, ion concentration changes, entry of infiltrating systemic components, and potential pathogens into the CNS may lead to neuronal dysfunction, neuroinflammation, and neurodegeneration^[34]^. The BBB plays a pivotal role in diseases associated with neuronal hyperexcitability such as epilepsy, TBI, and post-stroke seizure activity^[35–37]^. Microglia-neuron signaling had been shown initially by the release of the neuronal chemokine fractalkine, which activates the CXC-chemokine receptor 1 (CXCR1) on microglia. Neurogenesis, synaptic plasticity, and neuronal survival have all been reported to be affected by the CXCR1 signaling pathway^[31]^. Cytokine release of IL-1β and tumor necrosis factor-α (TNF-α) and other signals (such as HMGB1 and ATP) from activated astrocytes and microglia lead to hyperexcitability in neurons^[38,39]^. Precise targeting of reactive astrocytes and microglia for therapeutic intervention during epilepsy and epileptogenesis may be beneficial due to microglial involvement in the processes of neurogenesis, axonal sprouting, and neuroinflammation.

### Models of epilepsy

The pursuit of AEDs has provided > 30 medications, with many that were developed in the 1980s^[40]^. Although several animal models of epilepsy exist, clinically validated models, ones that are validated to predict efficacy and tolerability, are limited and currently only consist of three models: the maximal electroshock (MES) seizure protocol, subcutaneous pentylenetetrazol (scPTZ) acute seizure tests, and the kindled rodent model of chronic hyperexcitability^[41]^. Though not validated, multiple other animal models have been developed that have contributed to the understanding of the premise of new therapeutic options^[42]^. Still, newer drugs continue to have similar adverse events or side effects without exhibiting greater efficacy^[43]^. Variation in seizure models can result in acute or chronic seizure paradigms, differences in severity, or the intervening time until seizures start^[44]^. Acute models lack persisting changes, like a decrease in seizure threshold or spontaneous seizures. Chronic seizure models of epilepsy accommodate a period during which epileptogenesis takes place and may better represent human epilepsy^[45]^. Newer models, such as the post-SE model, kindling^[46]^, or genetic models, have become more extensively used due to their ability to result in spontaneous seizures. The kindling model, where repeated electrical stimulation leads to enhanced seizure susceptibility, is commonly utilized as it has been associated with seizure induced plasticity and provides a way to study such plasticity. Combining SRS with convulsive behavior or video-electroencephalogram (EEG) represents a more accurate epilepsy model, though it is not considered a clinically validated model for AED discovery.

The chemical induction of status epilepticus, usually by injection of kainic acid or pilocarpine^[47,48]^, can result in animals exhibiting SRSs days to weeks after SE, and allows for the determination of post-seizure changes in the brain neuropil. Models using chemoconvulsants and kindling have provided researchers with a way to study changes in mossy fiber sprouting, neurogenesis, and neuroinflammation post-seizure.

## MICROGLIA

Microglia, which make up approximately 10% of the brain’s cells, are the central nervous system’s primary form of immune defense. Originally thought to only serve immune response functions, they are now widely recognized to perform important functions that contribute to the development and maintenance of a healthy brain. Microglia are dynamic cells that survey their environment for injury or infection. Ramified microglia rapidly and constantly extend and retract their processes to assess the environment^[49]^. By evaluating their surroundings, microglia can actively participate in neurogenesis^[50,51]^, neurotrophic functions^[52]^, neuronal phagocytosis^[53]^, modulation of axonal processes^[54]^, synapse formation and pruning^[55–57]^. It has also been proposed that microglia aid in neurotransmitter clearance, specifically glutamate^[58]^, due to their upregulation of glutamate transporter GLT-1 in a cortical injury model^[59]^. Many of these functions however, are reported to be similarly performed by astrocytes.

### Microglial contribution to epileptogenesis

Models of epilepsy provide insight into neuronal and glial behavior post-seizure. Microglia sense the injury, and their activation cascade is initiated^[60,61]^ as they migrate to the region of insult, where they then remain activated for about 4–5 weeks post-seizure^[62]^, creating an inflammatory environment around the site of seizure onset. The extent and duration of microglial activation depends on the model used^[63]^. Most, though not all, chronic seizure models of epileptogenesis present a persistent inflammatory state in neural tissue^[64]^. After an inciting event, inflammatory cascades can either begin in the CNS, or be activated by molecules in the systemic circulation via breakdown of the BBB^[65]^. The seizure-induced activation of microglia can be visualized and followed non-invasively by positron emission tomography using ^11^C-PK11195, a radiolabeled TSPO (a selective translocator protein) that is expressed at low levels in the healthy CNS, but upregulated when neuroinflammation is initiated. Although TSPO does not distinguish between microglia and infiltrating macrophages^[66]^, its upregulation provides clear proof of the neuroinflammatory state of post-seizure CNS. Acute neuroinflammation is thought to contribute to chronic neuroinflammation states or worsen a pre-existing state^[67]^. Understanding how and when microglia are activated after seizures, and how they contribute over time to neuroinflammation may provide a target for downregulating or attenuating epileptogenesis.

Cytokines are signaling molecules that modulate inflammatory responses and are produced by neurons and glial cells after seizures. Interleukin-1β (IL-1β), IL-2, and IL-6 are present in the brain at low concentrations, which increase post-seizure^[68]^. Following seizures, mRNA expression of IL-1β, IL-6, TNF-α, TGF-β, and vascular endothelial growth factor (VEGF) were all reported to be upregulated ithe hippocampus. IL-1β may induce seizures by upregulating N-methyl-D-aspartate (NMDA) receptors on post-synaptic cells^[69]^. Studies also suggest that uncontrolled levels of IL-1β impair synaptic plasticity and cause neuronal dysfunction^[70]^. Other studies have demonstrated that IL-1β decreased GABA-mediated neurotransmission, leading to neuronal hyperexcitability and seizures^[71]^. When IL-1β activity was blocked, acute or recurrent seizures were reduced in rodent models^[38,72,73]^. Anakinra, a recombinant IL-1 receptor antagonist, was successfully used in a clinical study to treat febrile infection-related epilepsy syndrome (FIRES), demonstrating that IL-1β may be a crucial target in controlling seizure recurrence^[74]^. TNF-α is released by microglia and astrocytes, when low levels of glutamate are detected, to maintain neuronal excitation levels by upregulating synapses^[75]^. TNF-α also increases microglial glutamate release through glutaminase and gap junction regulation^[76]^ and regulates the adhesion molecule N-cadherin, which is involved in the organization of synapses^[77]^. Like IL-1β, TNF-α also affects GABA levels by increasing GABA receptor endocytosis, reducing its inhibitory action^[78]^. Another pro-inflammatory cytokine, IL-6, is upregulated by TNF-α and IL-1β. IL-6 has been reported to decrease hippocampal neurogenesis while increasing microgliosis, possibly contributing to epileptogenesis^[79]^.

### Changes in microglia post-seizure

The question of microglial activation status and its effects post-seizure have yet to be answered. Microglia modulate the severity of early seizures in a pilocarpine model with lipopolysaccharide (LPS) preconditioning^[80]^: ablation of microglia prior to seizure onset resulted in dramatic increases of seizure severity. Since no other cell types were affected by the method of microglia ablation^[81,82]^, it is suggested that microglia may play a role early on in seizure induction to protect the CNS from exaggerated neuronal activity. The presence of microglia may thus be beneficial during seizure; however, evidence suggests that their activation may be detrimental post-seizure. Minocycline, a tetracyclic antibiotic that has anti-inflammatory properties, has been shown to act as an inhibitor of microglial proliferation/activation^[83]^. Studies that used minocycline have reported that it protects against neuronal cell death after seizures, thus indicating that microglia contribute to neurodegeneration following seizures^[84]^. Other studies demonstrated that a 2-week course of minocycline post-status epilepticus decreased the number, duration, and severity of spontaneous recurrent seizures, suggesting that microglia are involved in the propagation of these SRS^[85–87]^. It should also be noted, on the other hand, that there are studies that show only partial effectiveness by minocycline^[88]^, or inability to reverse the increase of epileptogenesis^[89,90]^.

Inflammatory cytokines increase neuronal excitability and are believed to contribute to epileptogenesis^[91]^. Though inflammatory cytokines are expressed by several cell types in the brain, microglia-specific pro-inflammatory cytokines, such as IL-1β, IL6 and TNF-α, showed increased expression three days after SE but had diminished by day 21^[63]^. Levels of anti-inflammatory cytokines, such as Arg1, IL-4 and IL-10, were also increased. These data contribute to the existing controversy on the role that microglia and cytokines play post-seizure. Additionally, Toll-like receptor (TLR) signaling has been implicated in the production of cytokines in seizure models. Studies have demonstrated that the downregulation of TLR3 and TLR4 activities reduces recurrent and acute seizures, respectively^[92,93]^. Another study showed that the activated TLR4 pathway (mediated by MyD88) was part of the molecular response contributing to a pro-inflammatory environment post-SE^[94]^. Matsuda *et al*.^[95]^ reported that microglia secrete TNF-α to decrease the proliferation of neural progenitor cells (NPCs) in the subgranular zone (SGZ) and demonstrated that microglial activation is partly mediated through TLR9 post-SE. These studies emphasize the need for a better understanding of the role of cytokine signaling post-seizure.

## NEUROGENESIS

Neurogenesis, the incorporation of new neurons into the hippocampus, is a controlled process that affects fundamental brain activities such as memory formation and learning. Neurogenesis, and the newborn cells generated, contribute to brain plasticity and can be followed through maturation using specific markers. The progression from newborn cells to mature neurons can be tracked using markers such as Nestin and Sox-2 for newborn cells, doublecortin and polysialylated neuronal cell adhesion molecule for immature neural progenitor cells, and NeuN for mature neurons^[96]^. In recent years, there has been an increased effort to determine some of the major regulators of the neurogenic process in the adult brain^[97–99]^. Neurogenesis, mediated by the activation and differentiation of adult neural stem cells (NSCs), has been documented to occur primarily in two regions of the adult CNS: the subventricular zone (SVZ) of the lateral ventricles, and within the SGZ of the dentate gyrus (DG) in the hippocampus^[100,101]^. Neurogenesis in the hippocampus will be the main focus of this section, as the hippocampal region has been intimately linked and affected by seizures and epilepsy.

In rodent models of neurogenesis, radial glia-like NSCs located in the SGZ give rise to NPCs^[102]^. The neurogenic process involves five intricate stages, ultimately leading to the integration of newly mature granule cells in the hippocampus. During the first stage, NSCs proliferate and generate neural progenitors in the SGZ. Stage 2 is the continuous phase of survival, where NSC and progenitor cells are lost through apoptosis, in this early part of the process. During stage 3, progenitor cells undergo fate determination and differentiate into immature neurons. In stage 4, immature neurons migrate a short distance within the granule cell layer where they continue their maturation and integrate (Stage 5) into the hippocampal circuitry, receiving input from the entorhinal cortex, and projecting axons to the CA3 (mossy fibers) and hilar regions of the hippocampus^[101,103–106]^, which further synapse with CA1 pyramidal cells^[107]^.

In epilepsy, while the stimuli to trigger adult neurogenesis are activated, the orchestrated differentiation process is dysregulated at various steps. The newly formed granule neurons do not integrate appropriately into the dentate gyrus, thus forming aberrant connections with other neuronal cells, and contributes to epilepsy and associated cognitive decline^[108–110]^.

### The role of microglia in physiological neurogenesis

Variations in neurogenesis properties from the embryonic stages to adulthood have been studied and show that newborn neuron populations decrease with age^[111]^, potentially due to a lowered ability of NSCs to regenerate^[112]^, or changes in environmental cues in the hippocampus, including an activated state of microglia^[113]^. Microglia have been shown to participate in neurogenesis, during multiple stages of the process through the contribution of factors that affect the proliferation and survival of NSCs^[114,115]^. Cognitive decline has been correlated with decreased neurogenesis^[116]^, and studies provide support to the idea that exercise or enriched environments result in an increase in neurogenesis^[117–119]^, which may be modulated by microglial activation^[120]^. A pro-inflammatory environment has been demonstrated to inhibit adult neurogenesis, while anti-inflammatory treatments were able to rescue the phenotype^[121,122]^. All these findings demonstrate the need to understand the role of microglia in neurogenesis that takes place in the physiological and pathological CNS. The function of microglia is most likely influenced by the environmental signals in a particular setting, which will dictate the direction of their activation status.

Microglia constantly survey their environment and are in the proximity of all cell types during neurogenesis, including newborn neurons. They are also involved in the phagocytosis of NPCs and neuroblasts in a homeostatic role for maintaining neurogenic stem cells without releasing pro-inflammatory cytokines^[51]^. In concordance with these data, ablating microglia in the DG inhibited adult neurogenesis by diminishing neuroblast survival^[123]^. Although these effects are most likely mediated by the secretion of cytokines and by microglial-regulated phagocytosis, the influence of microglia on neurogenesis also extends beyond these molecular steps and events. There is a growing body of evidence demonstrating that microglial receptors can modulate their activity in neurogenesis. For example, microglial P2Y13 receptor was recently described to contribute to microglial structural integrity. When the P2Y13 receptor is knocked out, increases in proliferation of NPCs and new neurons are observed, and this may be another way to regulate neurogenesis^[124]^. CX3CR1 has also been demonstrated to be involved in the regulation of adult neurogenesis: microglia have been reported to activate NPCs through CX3CR1 pathways in the hippocampus^[125]^, and CX3CR1 null (−/−) mice exhibited impaired connectivity and aberrant synapse formation^[126]^. This was further supported by genetic and pharmacological inhibition of CX3CR1 signaling, which also led to aberrant neurogenesis^[127,128]^.

Abundant data show that microglia are critical in adult neurogenesis and regulate several stages of accurate incorporation of new neurons into the hippocampal circuitry. As several seizure disorders and models manifest predominantly in the hippocampus, the effects of epileptic activity on SGZ neurogenesis is starting to be uncovered.

### Neurogenesis and the pathophysiology of epilepsy

**Figure 2.**
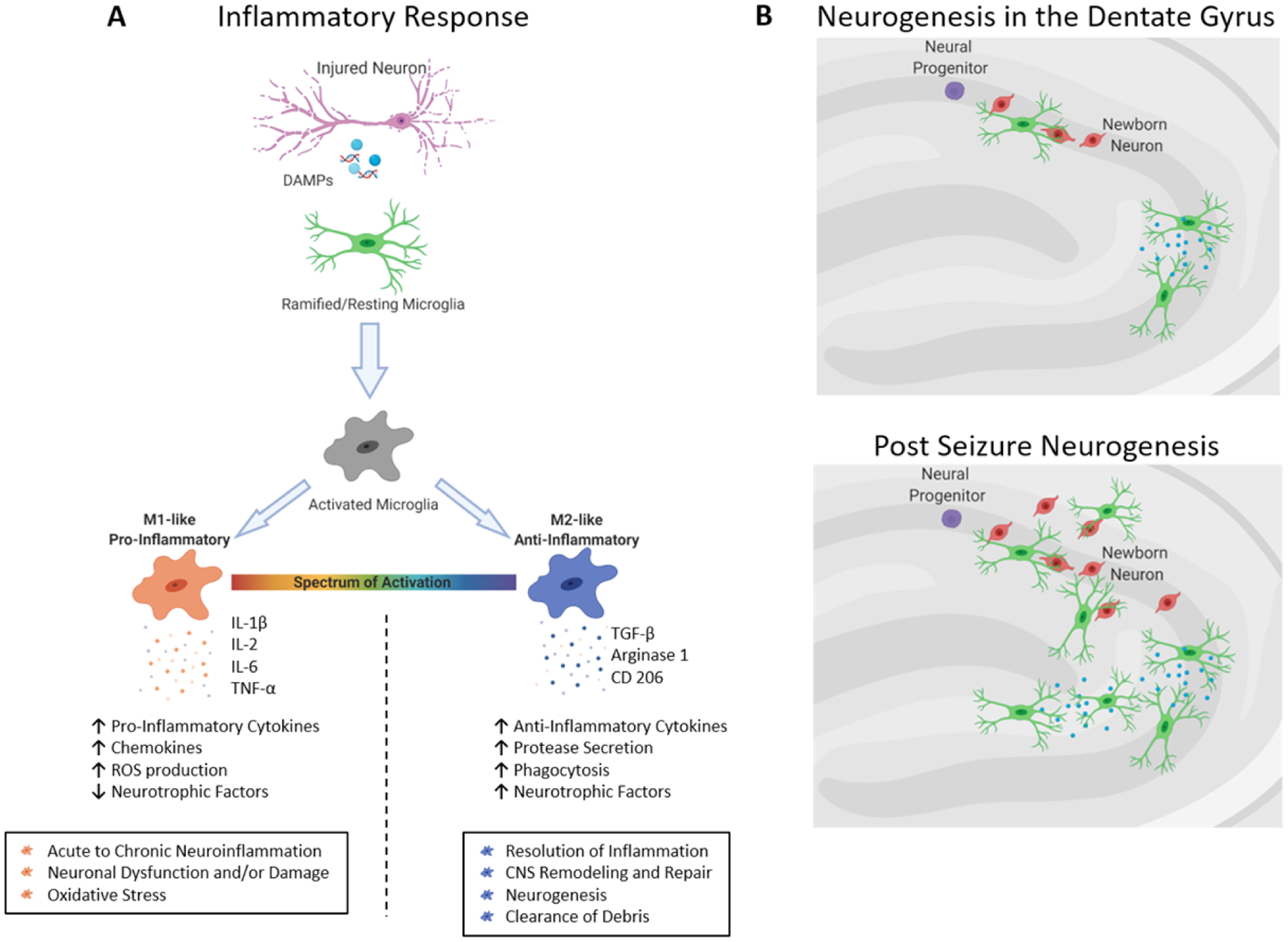
Microglial responses in fnflammation and neurogenesis. A: Microglia activate in response to damage associated molecular patterns (DAMPs) released by injured neurons post-seizure. Upon activation, microglial adopt one of two phenotypes: M1-like, which presents a pro-inflammatory profile that consists of decreased expression of neurotrophic factors and increased levels of pro-inflammatory chemokines and cytokines and reactive oxygen species, or M2-like, which is an anti-inflammatory response that includes the resolution of the inflammatory profile, neurogenesis and the clearance of debris; B: during neurogenesis in the hippocampus, unchallenged microglia clear cellular debris and control the number of newborn neuronal cells through phagocytosis. Post-seizure, the increased numbers of newborn cells may be cleared by microglia to reduce the potential for ectopic connections that contribute to pro-epileptic activity. Image created with Biorender.com

Adult neurogenesis increases following SE in animal models, resulting in an increased number of granule cells^[129,130]^. These additional granule cells undergo aberrant differentiation, axonal sprouting, and ectopic displacement in the hilar region of the dentate gyrus^[109,131,132]^. Ectopic granule cells are thought to contribute to pro-epileptic activity^[133–135]^; studies show that axonal sprouting and aberrant placement of granule cells were reduced when newborn granule cells were eliminated^[132]^. Following SE, microglia regulate the number of new granule cells through selective phagocytosis to maintain homeostasis in the dentate gyrus circuitry^[136]^ and are capable of engulfing viable neurons in the hippocampus as well^[137]^. It has been suggested that microglia modulate each step (proliferation, survival, and maturation) of adult neurogenesis in both homeostasis and epileptic states^[138]^, though their exact role in the integration of new cells has not been elucidated. Microglia may also suppress aberrant neurogenesis through the secretion of TNF-α^[95]^, potentially leading to anti-epileptic effects [Figure 2]. Recent studies depleting microglia from the SVZ suggested that they might not be necessary for NSC proliferation^[139,140]^, although this has not been shown in the hippocampus.

## CONCLUSION

Investigation of inflammatory and neurogenic processes in epilepsy has revealed potential and critical roles of microglia in several facets of seizure generation. Epilepsy patients take AED with the aim of preventing seizures, yet studies looking at the anti-inflammatory and neurogenic effects of these drugs are sparse. Interrogating the literature for effects of AEDs in vivo on microglia, an important modulator of these processes, result in surprisingly few reports^[141–143]^.

In vitro studies on microglial cells as mediators of inflammation have demonstrated that topiramate, a second generation AED, decreased the release of IL-1β, IL-6 and TNF-α^[144]^. Other AEDs such as levetiracetam, gabapentin, and phenobarbital showed slight modification in cytokine production^[145]^. The first generation AED valproic acid, was shown to increase IL-6 and TNF-α production in LPS-induced microglial cells^[145]^, which contrasts with in vivo results where TNF-α and IL-1β were decreased after valproic acid treatment^[143]^. It was also demonstrated that the AED levetiracetam suppressed neuroinflammation and phagocytosis in a pilocarpine induced SE model^[143]^. Itoh *et al*.^[146]^ reported that levetiracetam lessened microglial activation, as demonstrated by lower numbers of Iba-1 positive microglia, higher ramified shape, and low expression of pro-inflammatory cytokines. While the results of in vitro studies may eventually be applicable to the clinic, they highlight the need for clarification of the effects of AEDs on inflammation in vivo.

Studies concerning AEDs and neurogenesis are also extremely limited. Pregabalin, a widely used AED with an unknown mechanism of action, has been shown to accelerate the maturation of granule cells in the dentate gyrus^[147]^. In rats, lamotrigine increased the number of newborn cells in the hippocampus^[148]^ and increased neurogenesis^[149]^. Valproic acid also induced neurogenesis, but these effects were not induced by phenobarbital and topiramate^[149]^.

Epileptogenic changes in the brain are provoked by inflammation and increased neurogenic levels post-seizure. To control this process, a greater understanding of microglial contributions is needed and could provide a mechanism and target for a new generation of AEDs.
